# Endoscopic Cauterization of the Sphenopalatine Artery to Control Severe and Recurrent Posterior Epistaxis

**Published:** 2013-06

**Authors:** Behrooz Gandomi, Mohammad Hosein Arzaghi, Bijan Khademi, Mohammad Rafatbakhsh

**Affiliations:** 1 *Department of Otorhinolaryngology, Shiraz University of Medical Sciences, Shiraz, Iran*.; 2 *English Language Lecturer, Language Department, Shiraz University of Medical Sciences, Shiraz, Iran.*

**Keywords:** Epistaxis, Endoscopic sphenopalatine artery cauterization, SPA electrocoagulation technique

## Abstract

**Introduction:**

Epistaxis is one of the most common medical emergencies, making the management of posterior epistaxis a challenging problem for the ear, nose, and throat (ENT) surgeon. In the cases of conservative management failure, ligation of the major arteries or percutaneous embolization of the maxillary artery is performed routinely in most units, but rates of failure and complications are high. The objective of this study was to assess the effectiveness of endoscopic sphenopalatine artery (SPA) cauterization in patients with refractory posterior epistaxis.

**Materials and Methods:**

Between April 2011 and January 2012, 27 patients (15 males and 12 females) with refractory posterior epistaxis underwent endoscopic SPA cauterization in two tertiary referral hospitals in Shiraz. Three patients underwent bilateral cauterization.

**Results:**

Four patients (from 30 arteries) had new epistaxis after surgery, three experienced subsequent epistaxis requiring medical treatment, and one patient had a minor epistaxis not needing treatment.

**Conclusion:**

The SPA electrocoagulation technique seems to be safe, simple, fast, and effective with low rates of morbidity and complications for the management of refractory posterior epistaxis. Endoscopic SPA cauterization should be considered as an immediate second-line management when conservative treatment as first-line management fails.

## Introduction

Epistaxis is one of the most common emergencies following an accident, and is therefore a major challenge for emergency departments. The estimated incidence is 1/1000 population per year (1). Epistaxis can be life- threatening due to aspiration, hypotension, and anemia as well as associated co-morbidities. Five-to-fifteen percent of patients requiring hospital admission for this condition will need some form of surgical intervention (1,2).

Up to 90% of epistaxis cases have their origin in the Kiesselbach area and are managed with chemical cautery or packing, but 10% of cases originate from the posterior nasal area and require more aggressive blockage or other interventions (3). Posterior nasal packing, including balloon tamponade, has a high failure rate, ranging from 26% to 52% (1,4,5).

Intractable epistaxis remains a challenge for otolaryngologists. Historically, internal maxillary artery ligation via a transantral approach and ligation of the ethmoidal vessels and the external carotid artery have been the treatment of choice when conservative management failed.

Over the past decade, with the widespread popularization of endoscopic sinus surgery and the deeper understanding of local regional anatomy, endoscopic control of the sphenopalatine artery (SPA) has been advocated as an effective alternative for the control of posterior epistaxis (1,6).

## Materials and Methods

A prospective study was carried out between April 2011 and January 2012. Patients with refractory posterior epistaxis underwent endoscopic SPA cauterization in two tertiary referral hospitals in Shiraz (Khalili and Dastgheib Hospitals). All patients had suffered intractable posterior epistaxis despite undergoing conservative management with anterior and posterior nasal packing. Informed consent was provided by all patients. Patients with any bleeding diatheses, including those receiving anticoagulant therapy, were excluded from our study. The assessment of late outcomes was performed through clinical visits and/or telephone contact. The mean time of follow-up was 6.2 months (range, 2–10 months). Patients were admitted at an average of 1.2 days before implementation of the intended procedure and were discharged an average of 1.6 days after surgery (Table. 1).

**Table 1:** Average Length of Hospital Stay and Follow-Up Period

Parameter	Mean	Range
**Age (years)**	45.3	17–78
**Follow-up (months)**	6.2	2–10
**Pre-op stay (days)**	1.2	1–2
**Post-op stay (days)**	1.6	1–3

**Fig 1:**
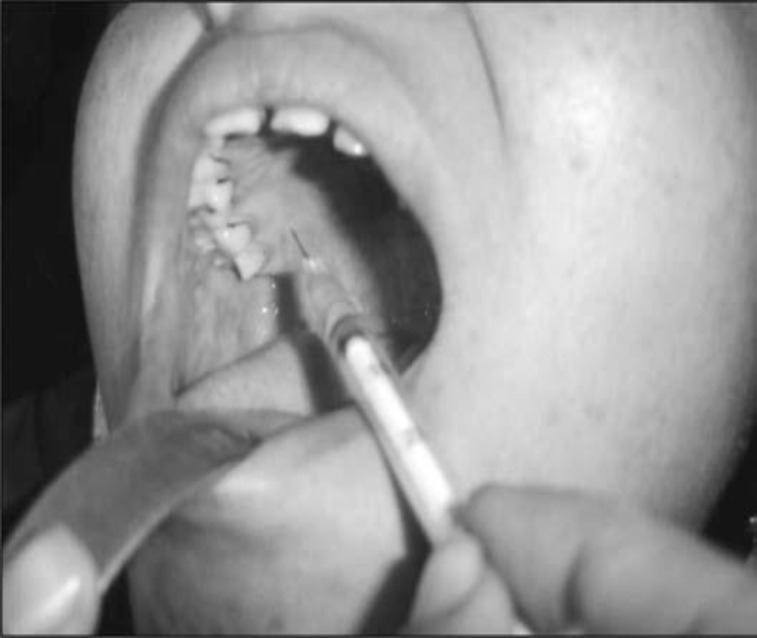
Injection into the pterygopalatine fossa through greater palatine foramen

The procedure was performed under general anesthesia. Initially, the nasal packing was removed and xylocain 2% (1 mL) with I:80,000 adrenaline was injected into the pterygopalatine fossa through the greater palatine foramen in the roof of the hard palate (Fig.1). Once the bleeding was controlled and the nose decongested, the membranous posterior fontanelle of the maxillary sinus was located by palpating under the bullae ethmoidalis. The junction between the membranous posterior fontanelle and the lateral nasal wall was then spotted. An incision was made from the undersurface of the horizontal portion of the ground lamella to the insertion point of the inferior turbinate on the lateral nasal wall. The mucoperiosteal flap was elevated using a Freer elevator and then dissected posteriorly, creating a mucosal tunnel under the sphenopalatine neurovascular bundle. The SPA trunk or branches were then spotted, being posterior to the ethmoidal crista (Fige.2). The SPA was then cauterized with bipolar diathermy, either as a single trunk or as its posterior lateral and posterior septal branches, depending on the individual anatomy. Finally, the mucoperiosteal flap was repositioned, and the nasal cavity was packed. The nasal pack was typically removed one day after surgery.

**Fig 2:**
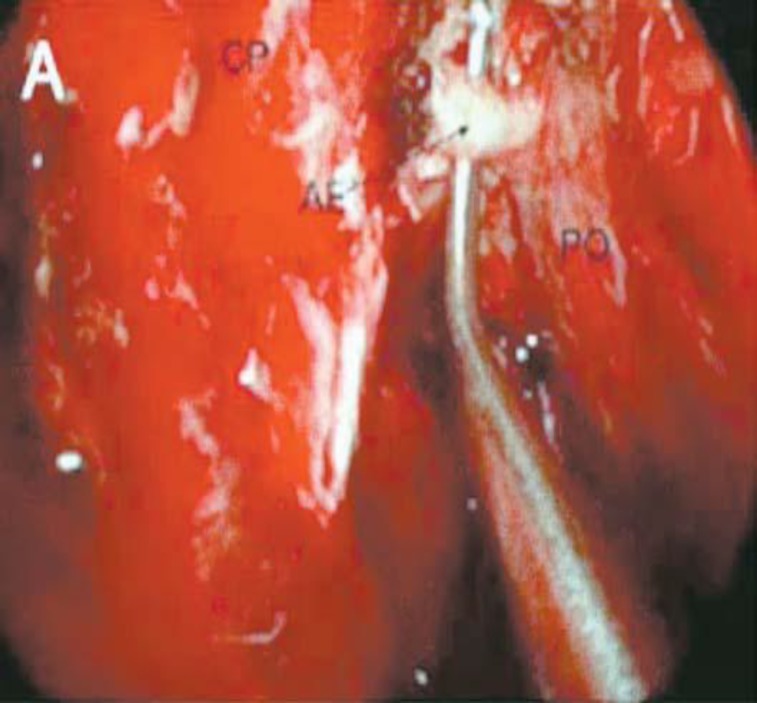
Sphenopalatine artery

## Results

**Fig 3:**
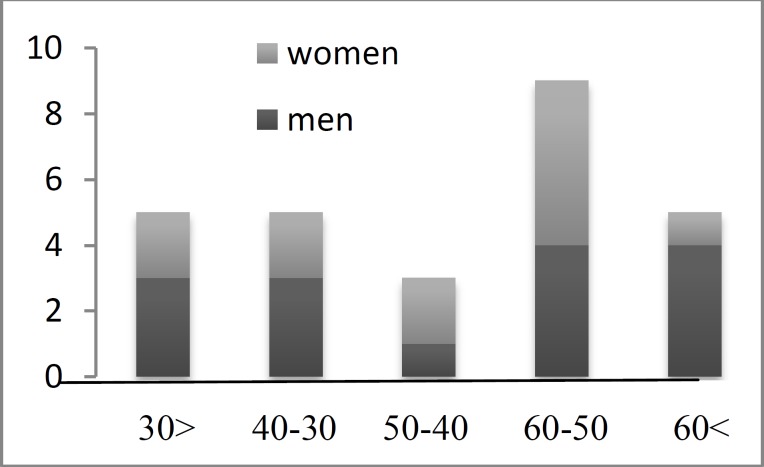
Age range of patients

Twenty seven patients (15 men and 12 women) underwent surgery on 30 arteries. Three patients required bilateral cauterization. The mean age of the patients was 45.3 years, ranging from 17 to 78 years (Fig.3). All operations involved cauterization with bipolar diathermy. The patients were later followed-up for an average of 6.2 months (range, 2–10 months).

No patient suffered recurrent epistaxis within the first 24 hours of surgery (immediate post-op period). Three patients suffered recurrent epistaxis within 2 weeks following surgery (early post-op period), two of whom needed anterior nasal packing with the third requiring no medical attention. One further patient had recurrent epistaxis within 7 days (early post-op period) and also 2 months after surgery (late post-op period). This patient further responded to conservative management including local cautery and anterior nasal packing. None of these four patients required any new surgical intervention (Table2). Therefore, the success rate in our study is 87%. 

**Table 2:** Epistaxis Recurrence

Timing	Patients (n)	Timepoints
**Immediate post-op** **period (24 hrs)**	0	-
**Early post-op** **Period (24 hrs–2 weeks)**	3	Days 7, 8, 10
**Late post-op** **Period (>2 weeks)**	1	2 months (also 7 days)

## Discussion

In recent years, with advances in surgical technology, the advent of endoscopic sinus surgery has revolutionized the treatment of sinus disease and has increased our knowledge of the anatomy of the lateral nasal wall. Management of epistaxis can be very difficult, especially when there is recurrent posterior epistaxis. Posterior nasal packing is a very inconvenient method for patients and carries a high failure rate (26–52%) (3), largely due to the fact that the turbinate prevents direct pressure on the bleeding point, causing the need for repeated blockage. Mucosal traumatism causes necrosis and further bleeding, starting a vicious circle. In addition, it has a high rate of complications (69%), including synechia, sinusitis, lesions in the nasal mucosa, local infections, septal perforation, orbital cellulitis, necrosis of the nasal ala, fracture of the lamina papyracea, perforation of the palate, and alterations in the middle ear. Other possible complications, although very rare, include pyogenic granuloma, allergy, toxic shock syndrome, obstructive sleep apnea syndrome (OSAS), hypoventilation, chest pain, hypoxia, aspiration (if the tamponade is moved), altered arterial blood gases, bradycardia, hypotension, and infectious endocarditis (3,22–25).

In the past, the most common approach to access the end artery of the lateral nasal wall was through the posterior wall of the maxillary sinus, using a Caldwell-Luc approach. Ligation of the internal maxillary artery (IMA) has its own considerable failure rate (10–13%), as well as an associated morbidity (14,26,27). Metson and Lane analyzed IMA failures and found that the most common cause was the surgeons' inability to locate the IMA or its terminal branches in the pterygomaxillary fossa (26). In addition, the opening of the anterior face of the maxillary sinus suggested by the Caldwell-Luc procedure can cause sinusitis, dental injury, infraorbital nerve damage, oro-antral fistulas, blindness, and ophthalmoplegia (20).

The management of intractable epistaxis involves angiography and the embolization of the IMA. Furthermore, the embolization of the maxillary artery demands the intervention of experienced radiologists. Metson and Lane, in their work on analyzing 100 failures of IMA ligation, noticed that the diverse branching patterns of the IMA frequently made vessel identification difficult. This fact may also be true for the SPA. However, they also found that in 13% of unsuccessful cases, the clips were not properly closed (12). Despite the high rate of success (75–90%) there is also a high rate of complications. The most common complic- ations are of neurological nature, and include hemiplegia, facial paresthesia, ophthalm- oplegia, blindness, and stroke. Ligation of the IMA has a success rate of approximately 90%, yet the rate of complications is approximately 30%.

One of the reasons that SPA ligation has become popular is that the SPA is the terminal branch of the IMA and is sufficiently distal to make retrograde and anastomotic blood flow from other vessels unlikely (29). This fact has led surgeons to consider how to better approach the SPA in an attempt to minimize both morbidity and failure rates (27,28,39), despite the description of transantral routes to the sphenopalatine artery (20).

The trans-nasal endoscopic approach, in which the artery is ligated while accessing the Vidian nerve, was first introduced in 1976 (20). With the endoscopic trans-nasal approach, the SPA can be easily reached. The SPA is the terminal branch of the IMA and is the dominant source of posterior nasal blood supply. Endoscopic SPA ligation is technically straightforward to perform, allowing direct and secure ligation of the major vessel supplying the posterior nasal cavity, while complications associated with pterygopalatine fossa surgery are avoided (1,6). This technique reduces the potential for intra-orbital and intracranial complications (1).

Over a period of 30 years (1982–2011), multiple studies have reported the success rates of ligation of the SPA (Table 3). Patients’ follow-up time varied between 6 to 36 months, and the rebleeding rate ranged from 3 to 30%. Rebleeding can be caused by various factors, including clip dislodgement, the presence of rich collateral vessels, and also the existence of more than one branch at the point of emergence of the SPA from its foramen which may partially account for this fairly consistent failure rate. Lee et al. found that the SPA was divided into two major branches in 76% of cases, into three branches in 22% of cases and into four branches in 2% of cases (16). It is to be noted that the success rate ranged between 70–100%. The majority of studies did not report any post-operative complications; although in one study, 53% of patients developed complications. Crust formation, a sensation of dryness in the nose, and persistent posterior rhinorrhea were the most frequent complications (3). Asanua et al. performed a retrospective study to compare bilateral endoscopic ligation of the SPA with that of the SPA accompanied by bilateral external ligation of the anterior ethmoidal artery in the management of persistent epistaxis (21). No statistically significant difference was noticed between the two groups in this regard.

Our study aimed at determining the advantages of SPA ligation over other methods and assessing the immediate and late post-operative cessation of epistaxis as well as complications associated with the procedure.

The ligation of the SPA leads to considerable complications. In some studies, 33% of cases had increased nasal crusting following this procedure. Also palatal numbness was reported in 13 % of cases. However, in our study, no patient reported any of these complications. Sphenopalatine artery ligation has been shown to be associated with a shorter hospital stay and cost effectiveness compared with other surgical modalities (1). There are no contraindications for SPA cauterization. Thus we recommend the use of this procedure for the control of posterior epistaxis as an immediate second-line management when conservative treatment as first line fails.

**Table 3:** Success rate of SPA ligation in other studies

**Study**	**Patients (n)**	**F/U (months)**	**Success (%)**	**Complication(%)**
Abdel Kader (1)	45	Up to 36	87	0
El Guindy (8)	9	3–6	100	0
Holzmann (9)	75	2–18	96	16
O'Flynn (10)	12	9	88	0
Rasmussen (11)	16	Up to 3	83	0
Rockey (12)	17 (1+AEA)	Up to 1	70	-
Rudert (13)	31	23	97	0
Sharp (14)	11	9	100	0
Simpson (15)	14	4–36	93	7
Snyderman (16)	42	1–28	88	53
Srinivasan (17)	10 (4+AEA)	10	90	0
Tsai (18)	9	2–14	95	0
Wiorowski (19)	10 (4+AEA)	1	90	0
Wormald (20)	13	5–28	92	0

AEA: Anterior ethmiodal artery

## Conclusions

Endoscopic cauterization of SPA is technically straightforward to perform and allows direct cauterization of the major vessel supplying the posterior nasal cavity. The procedure appears to be safe, simple, fast, and effective for the management of refractory posterior epistaxis with low rates of morbidity and complications, and should be considered as an immediate second-line management when conservative treatment as first line fails. Some experts even recommend its use as a first option, without waiting for the failure of posterior packing (3).
